# Supplementary material for: A Review about the Mycoremediation of Soil Impacted by War-like Activities: Challenges and Gaps
Source: J Fungi (Basel). 2024 Jan 24;10(2):94. doi: 10.3390/jof10020094 (PMC10890077; doi:10.3390/jof10020094)
Supplement: Supplementary file 1 [file jof-10-00094-s001.zip › jof-2752737-supplementary.pdf]

SUPPLEMENTARY MATERIALS

A Review about the Mycoremediation of Soil Impacted by War-like Activities: Challenges and Gaps

Regina Geris [1], Marcos Malta [1], Luar Aguiar Soares [2], Lourdes Cardoso de Souza Neta [2], Natan Silva Pereira [2], Miguel Soares [1], Vanessa da Silva Reis[2] and Madson de Godoi Pereira [2]*

[1] Institute of Chemistry, Federal University of Bahia, Barão de Jeremoabo Street, s/n, Campus Ondina, Zip Code: 40170-115, Salvador-BA, Brazil.

[2] Department of Exact and Earth Sciences - Bahia State University, Silveira Martins Street, N. 2555, Cabula, Zip Code: 41150-000, Salvador-BA, Brazil.

* Corresponding author: madson.pereira444@gmail.com and mpereira@uneb.br

Table S1. Experimental conditions for cultivating fungi used to remove explosives, metalloids, radionuclides, and herbicides from different media.

Pollutants	Fungal Species	Experimental Conditions	Ref.
**RDX**			
	**White Rot Fungi**		
	Phanerochaete chrysosporium	RDX was added after six days of culture, and cultures were incubated for an additional 30 days at 39°C.<br><br>Fungus was grown in 10 g soil contaminated with RDX, and cultures were incubated for an additional 30 days at 39°C.	[175, 176]
	Phanerochaete chrysosporium	RDX was added after two days in shake culture, and cultures were incubated for an additional 72 h.	[176, 177]
	Cyatus pallidum	RDX was added after two days in shake culture, and cultures were incubated for an additional 72 h.	[177]
	**Micromycetous fungi**		
	Cunninghamella echinulate	RDX was added after two days in shake culture, and cultures were incubated for an additional 72 h.	[177]
	Cladosporium resinae	RDX was added after two days in shake culture, and cultures were incubated for an additional 72 h.	[177]
**TNT**			
	**Wood-decaying basidiomycetes**		
	Fomes fomentarius[*]	After the fourth day of culture, TNT was added, and cultures were incubated at 140 rpm for an additional six days at 24°C in the dark.	[178]
	Heterobasidion annosum	After the fourth day of culture, TNT was added, and cultures were incubated at 140 rpm for an additional six days at 24°C in the dark.	[178]
	Hypholoma fasciculare	After the fourth day of culture, TNT was added, and cultures were incubated at 140 rpm for an additional six days at 24°C in the dark.	[178]
	Kuehneromyces mutabilis	After the fourth day of culture, TNT was added, and cultures were incubated at 140 rpm for an additional six days at 24°C in the dark.	[178]
	Laetiporus sulphureus	After the fourth day of culture, TNT was added, and cultures were incubated at 140 rpm for an additional six days at 24°C in the dark.	[178]
	Lentinula edodes	After the fourth day of culture, TNT was added, and cultures were incubated at 140 rpm for an additional six days at 24°C in the dark.	[178]

	Panus tigrinus	After the fourth day of culture, TNT was added, and cultures were incubated at 140 rpm for an additional six days at 24ºC in the dark.	[178]
	Phellinus robustus	After the fourth day of culture, TNT was added, and cultures were incubated at 140 rpm for an additional six days at 24ºC in the dark.	[178]
	Pleurotus abellatus	After the fourth day of culture, TNT was added, and cultures were incubated at 140 rpm for an additional six days at 24ºC in the dark.	[178]
	Pleurotus ostreatus	After the fourth day of culture, TNT was added, and cultures were incubated at 140 rpm for an additional six days at 24ºC in the dark.	[178]
	Trametes suaveolens	After the fourth day of culture, TNT was added, and cultures were incubated at 140 rpm for an additional six days at 24ºC in the dark.	[178]
	Trametes versicolor	After the fourth day of culture, TNT was added, and cultures were incubated at 140 rpm for an additional six days at 24ºC in the dark.	[178]
	Trametes versicolor<br>*Sclerotium rolfsii*	After the fifth day of culture, TNT was added, and cultures were incubated at 150 rpm for an additional four days at 30ºC.	[179]
	**Litter-decaying basidiomycetes**		
	Agaricus eastivalis	After the fourth day of culture, TNT was added, and cultures were incubated at 140 rpm for an additional six days at 24ºC in the dark.	[178]
	Agaricus bisporus	After the fourth day of culture, TNT was added, and cultures were incubated at 140 rpm for an additional six days at 24ºC in the dark.	[178]
	Agrocybe aegerita	After the fourth day of culture, TNT was added, and cultures were incubated at 140 rpm for an additional six days at 24ºC in the dark.	[178]
	Agrocybe praecox	After the fourth day of culture, TNT was added, and cultures were incubated at 140 rpm for an additional six days at 24ºC in the dark.	[178]
	Clitocybe odora	After the fourth day of culture, TNT was added, and cultures were incubated at 140 rpm for an additional six days at 24ºC in the dark.	[178]
	Coprinus comatus	After the fourth day of culture, TNT was added, and cultures were incubated at 140 rpm for an additional six days at 24ºC in the dark.	[178]
	Lepista nebularis	After the fourth day of culture, TNT was added, and cultures were incubated at 140 rpm for an additional six days at 24ºC in the dark.	[178]
	Paxillus involutus	After the fourth day of culture, TNT was added, and cultures were incubated at 140 rpm for an additional six days at 24ºC in the dark.	[178]
	Stropharia rugosoannulata	After the fourth day of culture, TNT was added, and cultures were incubated at 140 rpm for an additional six days at 24ºC in the dark.	[178]

	Stropharia rugosoannulata	Extracellular manganese peroxidase solution and TNT were incubated at 160 rpm for three days at 28°C in the dark.	[180, 181]
	**White Rot Fungi**		
	Bjerkandera adusta	After six days on malt extract broth culture, TNT was added, and cultures were incubated for an additional 12 days.	[182]
	Cyathus stercoreus	TNT was added to the growth medium, and cultures were incubated at 28°C for 28 days.	[183]
	Gymnopilus luteofolius	Mycelia were grown in the TNT soil contaminated* for 70 days.	[184]
	Irpex lacteus	After the fifth day of culture (YMG), TNT was added, and cultures were incubated at 130 rpm for an additional six days at 28°C.	[185]
	Nematoloma frowardii	Extracellular manganese peroxidase solution and TNT were incubated at 160 rpm for three days at 28°C in the dark.	[180, 181]
	Phanerochaete chrysosporium	After the fifth day of culture (YMG), TNT was added, and cultures were incubated at 130 rpm for an additional six days at 28°C.	[185]
	Phanerochaete chrysosporium	After six days of culture, TNT was added, and cultures were incubated for an additional 30 days at 39°C.<br><br>Fungus was grown in 10 g soil contaminated with RDX, and cultures were incubated for an additional 30 days at 39°C.	[175, 176]
	Phanerochaete chrysosporium	TNT was added to the growth medium, and cultures were incubated at 28°C for 28 days.	[183]
	Phanerochaete sordida	TNT was added to the growth medium, and cultures were incubated at 28°C for 28 days.	[183]
	Phanerochaete velutina	Mycelia were grown in the TNT soil contaminated* for 77 days.	[184]
	Phlebia brevispora	TNT was added to the growth medium, and cultures were incubated at 28°C for 28 days.	[183]
	Pleurotus ostreatus	After the fifth day of culture (YMG), TNT was added, and cultures were incubated at 130 rpm for an additional six days at 28°C.	[185]
	Pycnoporus coccineus	After the fifth day of culture (YMG), TNT was added, and cultures were incubated at 130 rpm for an additional six days at 28°C.	[185]
	Schizophyllum commune	After the fifth day of culture (YMG), TNT was added, and cultures were incubated at 130 rpm for an additional six days at 28°C.	[185]
	**Micromycetous fungi**		
	Alternaria sp.	After the fourth day of culture, TNT was added, and cultures were incubated at 140 rpm for an additional six days at 24°C in the dark.	[178]
	Aspergillus niger	After the fourth day of culture, TNT was added, and cultures were incubated at 140 rpm for an additional six days at 24°C in the dark.	[178]

	Aspergillus niger	Fungus was cultivated:<br>a) sterile soil for 30 days at 30°C,<br>b) non-sterile soil with native microorganisms and *A. niger* and *Mucor* sp.	[186]
	Aspergillus terreus	After the fourth day of culture, TNT was added, and cultures were incubated at 140 rpm for an additional six days at 24°C in the dark.	[178]
	Aspergillus sp.	Fungal discs in 8 mL of 68 mg L−1 TNT stock solution incubated at 25 °C, 125–130 rpm for 58 days.	[187]
	Cunninghamella elegans	After the fourth day of culture, TNT was added, and cultures were incubated at 140 rpm for an additional six days at 24°C in the dark.	[178]
	Fusarium oxysporum	After the fourth day of culture, TNT was added, and cultures were incubated at 140 rpm for an additional six days at 24°C in the dark.	[178]
	Fusarium solani	After the fourth day of culture, TNT was added, and cultures were incubated at 140 rpm for an additional six days at 24°C in the dark.	[178]
	Fusarium sp.	After the fourth day of culture, TNT was added, and cultures were incubated at 140 rpm for an additional six days at 24°C in the dark.	[178]
	Mucor mucedo	After the fourth day of culture, TNT was added, and cultures were incubated at 140 rpm for an additional six days at 24°C in the dark.	[178]
	Mucor sp.	Fungi were cultivated under Czapek´s medium, TNT added, and cultures were incubated at 200 rpm for 72h at 30°C.	[186]
	Mucor sp.	Fungi were cultivated:<br>a) sterile soil for 30 days at 30°C.<br>b) nonsterile soil with native microorganisms and *A. niger* and *Mucor* sp.	[186]
	Neurospora crassa	After the fourth day of culture, TNT was added, and cultures were incubated at 140 rpm for an additional six days at 24°C in the dark.	[178]
	Penicillium frequentans	After the fourth day of culture, TNT was added, and cultures were incubated at 140 rpm for an additional six days at 24°C in the dark.	[178]
	Penicillium sp.	After the fourth day of culture, TNT was added, and cultures were incubated at 140 rpm for an additional six days at 24°C in the dark.	[178]
	Rhizoctonia solani	After the fourth day of culture, TNT was added, and cultures were incubated at 140 rpm for an additional six days at 24°C in the dark.	[178]
	Rhizopus nigricans	TNT disappearance in malt extract broth.	[176]
	Thermomyces lanuginose	A composting system with a bacterial consortium of 55 °C agitated twice weekly for 91 days.	[176, 188]
	Trichoderma viride	Fungi were cultivated under Czapek´s medium, TNT added, and cultures were incubated at 200 rpm for 72h at 30°C.	[186]

	Trichoderma viride	After two days, 50 or 100 ppm TNT was added, and cultures were incubated for three days.	[189]
	Trichotecium sp.	Fungi were cultivated under Czapek´s medium, TNT added, and cultures were incubated at 200 rpm for 72h at 30ºC.	[186]
**Plutonium**			
	**White Rot Fungi**		
	Pleurotus ostreatus	Cultivation of oyster mushrooms in soil contaminated by solutions with 239Pu and 241Am radioactive isotopes.	[190]
**Uranium**			
	**Micromycetous fungi**		
	Aphanocladium spectabilis	The dry biomass was incubated in aqueous solutions containing uranium (VI) in a shaker operating at 160 rpm at varying final concentrations for 240 minutes.	[191]
	Acremonium minutisporum	The dry biomass was incubated in aqueous solutions containing uranium (VI) in a shaker operating at 160 rpm at varying final concentrations for 240 minutes.	[191]
	Aspergillus niger	Fungi were grown on a modified Czapek-Dox medium amended with glycerol 2-phosphate (G2P) as the sole P source and uranium nitrate.	[192]
	Gongronella butleri,	Live biomass was weighed (0.2 g) and added to sterile 50 mL falcon tubes containing 10 mL of uranium stock solution.	[193]
	Paecilomyces javanicus	Fungi were grown on a modified Czapek-Dox medium amended with glycerol 2-phosphate (G2P) as the sole P source and uranium nitrate.	[192]
	Penicillium piscarium, Penicillium citrinum, Penicillium ludwigii,	Live biomass was weighed (0.2 g) and added to sterile 50 mL falcon tubes containing 10 mL of uranium stock solution.	[193]
	Penicillium piscarium	Dead biomass was weighed (0.2 g) and added to 50 ml conical tubes containing 20 ml stock solutions with different pH and concentration (1, 10, 25, 50, and 100 mg L^{-1}).	[194]
	Talaromyces amestolkiae	Live biomass was weighed (0.2 g) and added to sterile 50 mL falcon tubes containing 10 mL of uranium stock solution.	[193]
**2,4-D**	**White Rot Fungi**		
	Pleurotus ostreatus	Fungus was inoculated in the medium and 2,4-D, and cultures were static incubated at 27 °C for 18 days.	[195]
	**Micromycetous fungi**		
	Aspergillus penicilloides	After two days of culture, 2,4-D was added, cultures were incubated for another five days at 24ºC, and the light was 1200 lux with a photoperiod of 12 h per day.	[196]
	Emericella nidulans	Fungus was inoculated in the PDB medium, and cultures were incubated at 20ºC for seven days,	[197]

		250 rpm. 50 mg of freeze-dried mycelium was incubated with 0.12 mM 2,4-D for 120h, 20°C. Fungus was cultivated in the culture medium containing 2,4-D for eight days, 250 rpm, 20°C in the dark.	
	Eupenicillium spp.	Rose Bengal liquid medium 2,4-D or 2,4,5-T after two weeks of incubation at 25 °C, a 1-mL aliquot was centrifuged (21 880 g, 10 min), and the supernatant was subjected to quantification.	[198]
	Fusarium sp.	After seven days of culture, 2,4-D was added, and cultures were incubated at 200 rpm for an additional seven days at 30°C.	[199]
	Mortierella isabellina	After two days of culture, 2,4-D was added, cultures were incubated for another five days at 24°C, and the light was 1200 lux with a photoperiod of 12 h per day.	[196]
	Penicillium miczynskii	Fungus was inoculated in the PDB medium, and cultures were incubated at 20°C for seven days, 250 rpm. 50 mg of freeze-dried mycelium was incubated with 0.12 mM 2,4-D for 120h, 20°C. Fungus was cultivated in the culture medium containing 2,4-D for eight days, 250 rpm, 20°C in the dark.	[197]
	Penicillium chrysogenum	Minimum mineral liquid medium with 2,4-D after two weeks of incubation at 30 °C, 160 rpm.	[200]
	Penicillium chrysogenum	The fungus was cultivated with 2,4-D on different media at 25 ± 1°C, 160 rpm.	[201]
	Rhizopus stolonifer	Minimum mineral liquid medium with 2,4-D after two weeks of incubation at 30 °C, 160 rpm.	[200]
	Rigidoporus sp.	After seven days of culture, 2,4-D was added, and cultures were incubated at 200 rpm for an additional seven days at 30°C.	[199]
	Talaromyces spp.	Rose Bengal liquid medium 2,4-D or 2,4,5-T after two weeks of incubation at 25 °C, a 1-mL aliquot was centrifuged (21 880 g, 10 min), and the supernatant was subjected to quantification.	[198]
	Trichoderma koningii,	Minimum mineral liquid medium with 2,4-D after two weeks of incubation at 30 °C, 160 rpm.	[200]
	Trichoderma viride	Minimum mineral liquid medium with 2,4-D after two weeks of incubation at 30 °C, 160 rpm.	[200]
	Umbelopsis isabellina	The cultivation was performed on a rotary shaker at 160 rpm for 24 h at 28 °C.	[202]
	Verticillium sp.	After seven days of culture, 2,4-D was added, and cultures were incubated at 200 rpm for an additional seven days at 30 °C.	[199]
**2,4,5-T**			
	**Micromycetous fungi**		
	Eupenicillium sp. VN 5-2-2-	Rose Bengal liquid medium 2,4-D or 2,4,5-T after two weeks of incubation at 25 °C, a 1-mL aliquot was centrifuged (21 880 g, 10 min), and the supernatant was subjected to quantification.	[198]

	Eupenicillium sp. VN 10-2-2-	Rose Bengal liquid medium 2,4-D or 2,4,5-T after two weeks of incubation at 25 °C, a 1-mL aliquot was centrifuged (21 880 g, 10 min), and the supernatant was subjected to quantification.	[198]
	Fusarium sp.	After seven days of culture, 2,4,5-T was added, and cultures were incubated at 200 rpm for an additional seven days at 30°C.	[199]
	Rigidoporus sp.	After seven days of culture, 2,4,5-T was added, and cultures were incubated at 200 rpm for an additional seven days at 30°C.	[199]
	Verticillium sp.	After seven days of culture, 2,4,5-T was added, and cultures were incubated at 200 rpm for an additional seven days at 30°C.	[199]
**TCDD**			
	**White Rot Fungi**		
	Rigidoporus sp.	FMD21 was incubated by 28 days culture with a start concentration of 0.5 pg TEQ/μL TCDD.	[203]
**As**			
	**Micromycetous fungi**		
	Absidia spinosa	A 6-m-diameter plug was placed on the MEA plate with As, and the cultures were incubated for seven days, 25°C in the dark.	[204]
	Acidomyces acidophilus	Fungal was inoculated in LSM for 21 days at 25 °C, 110 rpm. Adsorption test was carried out with freeze-dried biomass and 20 mL at 25 °C on an orbital shaker at 120 rpm.	[205]
	Arthroderma benhsmiae	The fungus was cultivated in a medium amended with arsenic, and cultures were incubated at 27 °C at 120 rpm for the first ten days and then at stationary phase for the next 11 days.	[206]
	Aspergillus clavatus	The fungus was inoculated in SAB medium enriched with arsenic in pentavalent form as a solution of arsenic acid for 30 days.	[207]
	Aspergillus niger A	The fungus was inoculated in SAB medium enriched with arsenic in pentavalent form as a solution of arsenic acid for 30 days.	[207]
	Aspergillus niger B	The fungus was inoculated in SAB medium enriched with arsenic in pentavalent form as a solution of arsenic acid for 30 days.	[207]
	Aspergillus flavus	Biosorption: As (V) was added to the SAB medium, and cultures were incubated for 30 days.<br>Biovolatilization (BV): volatile arsenic metabolites from mycelial headspaces were captured after 29 days.	[208]
	Aspergillus nidulans	The fungus was cultivated in a medium amended with arsenic, and cultures were incubated at 27 °C at 120 rpm for the first ten days and then at stationary phase for the next 11 days.	[206]

	Aspergillus niger<br>*Aspergillus* spp.	The biosorption capacity of the immobilized fungal biomass on the Luffa sponge was assessed by adding arsenic (As III) in a growth medium and the immobilized sponge in a flask, which was incubated at 28 °C for 3–4 days in a rotary shaker.	[209]
	Aspergillus oryzae,	The fungus was cultivated in a medium amended with arsenic, and cultures were incubated at 27 °C at 120 rpm for the first ten days and then at stationary phase for the next 11 days.	[206]
	Aspergillus ustus<br>*Aspergillus* sp.	The fungi were inoculated on a liquid medium at 30°C for five days, and As was added to the cultures and incubated for four more days.	[210]
	Cephalotrichum nanum	A 6 m-diameter plug was placed on MEA plate with As, and the cultures were incubated for seven days, 25°C in the dark	[204]
	Emericella sp.	The fungus was cultivated in a medium amended with arsenic, and cultures were incubated at 27 °C at 120 rpm for the first ten days and then at stationary phase for the next 11 days	[206]
	Eupenicillium cinnamopurpureum	Biosorption: As in the pentavalent form, it was added to the SAB medium, and cultures were incubated for 30 days.<br>Biovolatilization (BV): volatile arsenic metabolites from mycelial headspaces were captured after 29 days.	[208]
	Fusarium oxysporum	The spore suspension was inoculated into the liquid PGP medium containing As(III). After the fungal biomass was cultivated at 25 °C, 140 rpm for five days	[211]
	Fusarium oxysporum	The fungus was inoculated into the PGP medium spiked with As(V) for 15 days	[212]
	Fusarium sp.	The fungus was cultivated in a medium amended with arsenic, and cultures were incubated at 27 °C at 120 rpm for the first ten days and then at stationary phase for the next 11 days	[206]
	Metarhizium marquandii	A 6 m-diameter plug was placed on MEA plate with As, and the cultures were incubated for seven days, 25°C in the dark	[204]
	Neosartorya fischeri	Biosorption: As in the pentavalent form, it was added to the SAB medium, and cultures were incubated for 30 days.<br>Biovolatilization (BV): volatile arsenic metabolites from mycelial headspaces were captured after 29 days.	[208]
	Neocosmospora sp.	The fungus was cultivated in a medium amended with arsenic, and cultures were incubated at 30 °C, 120 rpm for 21 days	[213]

	Penicillium janthinellum	The spore suspension was inoculated into the liquid PGP medium containing As (III). After the fungal biomass was cultivated at 25 °C, 140 rpm for five days	[211]
	Penicillium janthinellum	The fungus was inoculated into the PGP medium spiked with As (V) for 15 days	[212]
	Penicillium glabrum	The fungus was inoculated in an SAB medium enriched with arsenic in pentavalent form as a solution of arsenic acid for 30 days	[207]
	Penicillium sp.	The fungus was cultivated in a medium amended with arsenic, and cultures were incubated at 30 °C, 120 rpm for 21 days.	[213]
	Purpureocillium lilacinum	A 6m-diameter plug was placed on the MEA plate with As, and the cultures were incubated for seven days, 25 °C in the dark.	[204]
	Rhizomucor variabilis	The fungus was cultivated in a medium amended with arsenic, and cultures were incubated at 27 °C at 120 rpm for the first ten days and then at stationary phase for the next 11 days.	[206]
	Rhizopus sp.	The fungus was cultivated in a medium amended with arsenic, and cultures were incubated at 30 °C, 120 rpm for 21 days.	[213]
	Sterile mycelial strain FA-13	The fungus was cultivated in a medium amended with arsenic, and cultures were incubated at 30 °C, 120 rpm for 21 days.	[213]
	Talaromyces wortmannii	Biosorption: As in the pentavalent form, it was added to the SAB medium, and cultures were incubated for 30 days.<br>Biovolatilization (BV): volatile arsenic metabolites from mycelial headspaces were captured after 29 days)	[208]
	Talaromyces flavus	Biosorption: As in the pentavalent form, it was added to the SAB medium, and cultures were incubated for 30 days.<br>Biovolatilization (BV): volatile arsenic metabolites from mycelial headspaces were captured after 29 days.	[208]
	Talaromyces sp.	The fungus grown in PDB for seven days was washed with deionized water and transferred into Erlenmeyer flasks containing deionized water and various concentrations of As (III) or As (V) (1~25 mg L^{-1}).<br><br>Inactivated mycelium was obtained from fungal cultures grown in PDB that were dried in an oven at 80 °C for 24 h after harvesting	[214]
	Trichoderma asperellum	The spore suspension was inoculated into the liquid PGP medium containing As (III). After the fungal biomass was cultivated at 25 °C, 140 rpm for five days	[211]

	Trichoderma asperellum	The fungus was inoculated into the PGP medium spiked with As(V) for 15 days	[212]
	Trichoderma atroviride	The fungus was cultivated in a medium amended with As (III) at 28 °C for five days, 125 rpm	[215]
	Trichoderma sp.	The fungus was cultivated in a medium amended with arsenic, and cultures were incubated at 30 °C, 120 rpm for 21 days	[213]
	Trichophyton verrucosum	The fungus was cultivated in a medium amended with arsenic, and cultures were incubated at 27 °C at 120 rpm for the first ten days and then at stationary phase for the next 11 days	[206]
	Trichoderma viride	The fungus was inoculated in an SAB medium enriched with arsenic in pentavalent form as a solution of arsenic acid for 30 days	[207]

RDX = hexahydro 1,3,5-trinitro-l,3,5-triazine. TNT = 2,4,6-trinitro-toluene. * TNT soil contaminated: originating from a military storage area (provided by the Construction Establishment of Finnish Defense Administration). 2,4-D = 2,4-Dichlorophenoxyacetic acid 2,4,5-T = 2,4,5-Trichlorophenoxyacetic acid. Orange agent = 2,4-D + 2,4,5-T. TCDD = 2,3,7,8-tetraclorodibenzo-p-dioxina.

References

[175] Fernando, T.; Aust, S.D. Biodegradation of munition waste, TNT (2,4,6-trinitrotoluene), and RDX (Hexahydro-1,3,5-trinitro-triazine) by *Phanerochaete chrysosporium*. *ACS Symp. Ser.* **1991**, *468*, 214–232.

[176] Bennett, J.W. Prospects for Fungal Bioremediation of TNT Munition Waste. *Int. Biodeterior. Biodegrad.* **1994**, *34*, 21–34. https://doi.org/10.1016/0964-8305(95)00001-1.

[177] Bayman, P.; Ritchey, S.D.; Bennett, J.W. Fungal interactions with the explosive RDX (hexahydro-1,3,5-trinitro-1,3,3-triazine). *J. Ind. Microbiol.* **1995**, *15*, 418–423. https://doi.org/10.1007/bf01569968.

[178] Scheibner, E.; Hofrichter, M.; Herre, A.; Michels, J.; Fritsche, W. Screening for fungi intensively mineralizing 2,4,6-trinitrotoluene. *Appl. Microbiol. Biotechnol.* **1997**, *47*, 452–457. https://doi.org/10.1007/s002530050955.

[179] Nyanhongo, G.S.; Erlacher, A.; Schroeder, M.; Gübitz, G.A. Enzymatic immobilization of 2,4,6-trinitrotoluene (TNT) biodegradation products onto model humic substances. *Enzym. Microb. Technol.* **2006**, *39*, 1197–1204. https://doi.org/10.1016/j.enzmictec.2006.03.004.

[180] Scheibner, K.; Hofrichter, M. Conversion of aminonitrotoluenes by fungal manganese peroxidase. *J. Basic Microbiol.* **1998**, *38*, 51–59.

[181] Esteve-Núñez, A.; Caballero, A.; Ramos, J.L. Biological degradation of 2,4,6-trinitrotoluene. *Microbiol. Mol. Biol. Rev.* **2001**, *65*, 335–352. https://doi.org/10.1128/mmbr.65.3.335-352.2001.

[182] Eilers, A.; Rüngeling, E.; Stündl, U.M.; Gottschalk, G. Metabolism of 2,4,6-trinitrotoluene by the white-rot fungus *Bjerkandera adusta* DSM 3375 depends on cytochrome P-450. *Appl. Microbiol. Biotechnol.* **1999**, *53*, 75–80. https://doi.org/10.1007/s002530051617.

[183] Donnelly, K.C.; Chen, J.C.; Huebner, H.J.; Brown, K.W.; Autenrieth, R.L.; Bonner, J.S. Utility of four strains of white-rot fungi for the detoxification of 2,4,6-trinitrotoluene in liquid culture. *Environ. Toxicol. Chem.* **1997**, *16*, 1105–1110. https://doi.org/10.1002/etc.5620160603.

[184] Anasonye, F.; Winquist, E.; Räsänen, M.; Kontro, J.; Björklöf, K.; Vasilyeva, G.; Jorgensen, K.S.; Steffen, K.T.; Tuomela, M. Bioremediation of TNT contaminated soil with fungi under laboratory and pilot scale conditions. *Int. Biodeterior. Biodegrad.* **2015**, *105*, 7–12. https://doi.org/10.1016/j.ibiod.2015.08.003.

[185] Kim, H.Y.; Song, H.G. Comparison of 2,4,6-trinitrotoluene degradation by seven strains of white rot fungi. *Curr. Microbiol.* **2000**, *41*, 317–320. https://doi.org/10.1007/s002840010142.

[186] Kutateladze, L.; Zakariashvili, N.; Khokhashvili, I.; Jobava, M.; Alexidze, T.; Urushadze, T.; Kvesitadze, E. Fungal elimination of 2,4,6-trinitrotoluene (TNT) from the soils. *EuroBiotech J.* **2018**, *2*, 39–46. https://doi.org/10.2478/ebtj-2018-0007.

[187] Castellanos, J.; Rios-Velazquez, C.; Morales, F.; Miranda-Berrocales, V.; Liquet-Gonzalez, J.; Cortez, I.; Padilla, R.; Vega-Olivencia, C.A.; Hernández-Rivera, S.P. Cyclic voltammetry as a screening tool for the fungal degradation of 2,4,6-trinitrotoluene in aqueous media. *Int. J. Environ. Anal. Chem.* **2016**, *96*, 978–989. https://doi.org/10.1080/03067319.2016.1220003.

[188] Kaplan, D.L.; Kaplan, A.M. *Thermophilic biotransformations* of 2,4,6-trinitrotoluene under simulated composting conditions. *Appl. Environ. Microbiol.* **1982**, *44*, 757–760. https://doi.org/10.1128/aem.44.3.757-760.1982.

[189] Alothman, Z.A.; Bahkali, A.H.; Elgorban, A.M.; Al-Otaibi, M.S.; Ghfar, A.A.; Gabr, S.A.; Wabaidur, S.M.; Habila, M.A.; Ahmed, A. Bioremediation of Explosive TNT by *Trichoderma viride*. *Molecules* **2020**, *25*, 1393. https://doi.org/10.3390/molecules25061393.

[190] Galanda, D.; Mátel, L.; Strisovská, J.; Dulanská, S. Mycoremediation: The study of transfer factor for plutonium and americium uptake from the ground. *J. Radioanal. Nucl. Chem.* **2014**, *299*, 1411–1416. https://doi.org/10.1007/s10967-013-2909-9.

[191] Gargarello, R.; Cavalitto, S.; Di Gregorio, D.; Niello, J.F.; Huck, H.; Pardo, A.; Somacal, H.; Curutchet, G. Characterization of uranium(VI) sorption by two environmental fungal species using gamma spectrometry. *Environ. Technol.* **2008**, *29*, 1341–1348. https://doi.org/10.1080/09593330802327069.

[192] Liang, X.J.; Hillier, S.; Pendlowski, H.; Gray, N.; Ceci, A.; Gadd, G.M. Uranium phosphate biomineralization by fungi. *Environ. Microbiol.* **2015**, *17*, 2064–2075. https://doi.org/10.1111/1462-2920.12771.

[193] Coelho, E.; Reis, T.A.; Cotrim, M.; Mullan, T.K.; Corrêa, B. Resistant fungi isolated from contaminated uranium mine in Brazil shows a high capacity to uptake uranium from water. *Chemosphere* **2020**, *248*, 126068. https://doi.org/10.1016/j.chemosphere.2020.126068.

[194] Coelho, E.; Reis, T.A.; Cotrim, M.; Rizzutto, M.; Corrêa, B. Bioremediation of water contaminated with uranium using *Penicillium piscarium*. *Biotechnol. Prog.* **2020**, *36*, e30322. https://doi.org/10.1002/btpr.3032.

[195] Pereira, J.C.V.; Raza, G.; Jenske, G.; Pereyra, L.; Serbent, M.P. Removal of 2,4-D herbicide from aqueous solution by *Pleurotus ostreatus*. *Braz. J. Chem. Eng,* **2023**. https://doi.org/10.1007/s43153-023-00338-7.

[196] Vroumsia, T.; Steiman, R.; Seigle-Murandi, F.; Benoit-Guyod, J.L.; Groupe pour l'Etude du Devenir des Xénobiotiques dans l'Environment. Fungal bioconversion of 2,4-dichlorophenoxyacetic acid (2,4-D) and 2,4-dichlorophenol (2,4-DCP). *Chemosphere* **2005**, *60*, 1471–1480. https://doi.org/10.1016/j.chemosphere.2004.11.102.

[197] Benoit, P.; Barriuso, E.; Calvet, R. Biosorption characterization of herbicides, 2,4-D and atrazine, and two chlorophenols on fungal mycelium. *Chemosphere* **1998**, *37*, 1271–1282. https://doi.org/10.1016/s0045-6535(98)00125-8.

[198] Itoh, K.; Kinoshita, M.; Morishita, S.; Chida, M.; Suyama, K. Characterization of 2,4-dichlorophenoxyacetic acid and 2,4,5-trichlorophenoxyacetic acid-degrading fungi in Vietnamese soils. *FEMS Microbiol. Ecol.* **2013**, *84*, 124–132. https://doi.org/10.1111/1574-6941.12043.

[199] Nguyen, T.L.A.; Dao, A.T.N.; Dang, H.T.C.; Koekkoek, J.; Brouwer, A.; de Boer, T.E.; van Spanning, R.J.M. Degradation of 2,4-dichlorophenoxyacetic acid (2,4-D) and 2,4,5-trichlorophenoxyacetic acid (2,4,5-T) by fungi originating from Vietnam. *Biodegradation* **2022**, *33*, 301–316. https://doi.org/10.1007/s10532-022-09982-1.

[200] Bhosle, N.P.; Thore, A.S. Biodegradation of the Herbicide 2,4-D by Some Fungi. *Am.-Eurasian J. Agric. Environ. Sci.* **2016**, *16*, 1666–1671. https://doi.org/10.5829/idosi.aejaes.2016.1666.1671.

[201] Ferreira-Guedes, S.; Mendes, B.; Leitão, A.L. Degradation of 2,4-dichlorophenoxyacetic acid by a halotolerant strain of *Penicillium chrysogenum*: Antibiotic production. *Environ. Technol.* **2012**, *33*, 677–686. https://doi.org/10.1080/09593330.2011.588251.

[202] Nykiel-Szymanska, J.; Stolarek, P.; Bernat, P. Elimination and detoxification of 2,4-D by *Umbelopsis isabellina* with the involvement of cytochrome P450. *Environ. Sci. Pollut. Res.* **2018**, *25*, 2738–2743. https://doi.org/10.1007/s11356-017-0571-4.

[203] Dao, A.T.N.; Vonck, J.; Janssens, T.K.S.; Dang, H.T.C.; Brouwer, A.; de Boer, T.E. Screening white-rot fungi for bioremediation potential of 2,3,7,8-tetrachlorodibenzo-p-dioxin. *Ind. Crops Prod.* **2019**, *128*, 153–161. https://doi.org/10.1016/j.indcrop.2018.10.059.

[204] Ceci, A.; Spinelli, V.; Massimi, L.; Canepari, S.; Persiani, A.M. Fungi and Arsenic: Tolerance and Bioaccumulation by Soil Saprotrophic Species. *Appl. Sci.* **2020**, *10*, 3218. https://doi.org/10.3390/app10093218.

[205] Chan, W.K.; Wildeboer, D.; Garelick, H.; Purchase, D. Competition of As and other Group 15 elements for surface binding sites of an extremophilic Acidomyces acidophilus isolated from a historical tin mining site. *Extremophiles* **2018**, *22*, 795–809. https://doi.org/10.1007/s00792-018-1039-2.

[206] Singh, M.; Srivastava, P.K.; Verma, P.C.; Kharwar, R.N.; Singh, N.; Tripathi, R.D. Soil fungi for mycoremediation of arsenic pollution in agriculture soils. *J. Appl. Microbiol.* **2015**, *119*, 1278–1290. https://doi.org/10.1111/jam.12948.

[207] Urík, M.; Cernansky, S.; Sevc, J.; Simonovicová, A.; Littera, P. Biovolatilization of arsenic by different fungal strains. *Water Air Soil Pollut.* **2007**, *186*, 337–342. https://doi.org/10.1007/s11270-007-9489-7.

[208] Cernansky, S.; Urík, M.; Sevc, J.; Khun, M. Biosorption and biovolatilization of arsenic by heat-resistant fungi. *Environ. Sci. Pollut. Res.* **2007**, *14*, 31–35. https://doi.org/10.1065/espr2006.11.361.

[209] Tanvi, D.A.; Pratam, K.M.; Lohit, R.T.; Vijayalakshmi, B.K.; Devaraja, T.N.; Vasudha, M.; Ramesh, A.; Chakra, P.S.; Gayathri, D. Biosorption of heavy metal arsenic from Industrial Sewage of Davangere District, Karnataka, India, using indigenous fungal isolates. *SN Appl. Sci.* **2020**, *2*, 1860. https://doi.org/10.1007/s42452-020-03622-0.

[210] Kamal, N.; Parshad, J.; Saharan, B.S.; Kayasth, M.; Mudgal, V.; Duhan, J.S.; Mandal, B.S.; Sadh, P.K. Ecosystem Protection through Myco-Remediation of Chromium and Arsenic. *J. Xenobiotics* **2023**, *13*, 159–171. https://doi.org/10.3390/jox13010013.

[211] Feng, Q.F.; Su, S.M.; Zeng, X.B.; Zhang, Y.Z.; Li, L.F.; Bai, L.Y.; Duan, R.; Lin, Z.L. Arsenite Resistance, Accumulation, and Volatilization Properties of *Trichoderma asperellum* SM-12F1, *Penicillium janthinellum* SM-12F4, and *Fusarium oxysporum* CZ-8F1. *Clean-Soil Air Water* **2015**, *43*, 141–146. https://doi.org/10.1002/clen.201200566.

[212] Su, S.M.; Zeng, X.B.; Bai, L.Y.; Li, L.F.; Duan, R. Arsenic biotransformation by arsenic-resistant fungi *Trichoderma asperellum* SM-12F1, *Penicillium janthinellum* SM-12F4, and *Fusarium oxysporum* CZ-8F1. *Sci. Total Environ.* **2011**, *409*, 5057–5062. https://doi.org/10.1016/j.scitotenv.2011.08.039.

[213] Srivastava, P.K.; Vaish, A.; Dwivedi, S.; Chakrabarty, D.; Singh, N.; Tripathi, R.D. Biological removal of arsenic pollution by soil fungi. *Sci. Total Environ.* **2011**, *409*, 2430–2442. https://doi.org/10.1016/j.scitotenv.2011.03.002.

[214] Nam, I.H.; Murugesan, K.; Ryu, J.; Kim, J.H. Arsenic (As) Removal Using *Talaromyces* sp. KM-31 Isolated from As-Contaminated Mine Soil. *Minerals* **2019**, *9*, 568. https://doi.org/10.3390/min9100568.

[215] Morales-Mendoza, A.G.; Flores-Trujillo, A.K.I.; Ramírez-Castillo, J.A.; Gallardo-Hernández, S.; Rodríguez-Vázquez, R. Effect of Micro-Nanobubbles on Arsenic Removal by *Trichoderma atroviride* for Bioscorodite Generation. *J. Fungi* **2023**, *9*, 857. https://doi.org/10.3390/jof9080857.
